# The deployment of balanced scorecard in health care organizations: is it beneficial? A systematic review

**DOI:** 10.1186/s12913-021-07452-7

**Published:** 2022-01-13

**Authors:** Faten Amer, Sahar Hammoud, Haitham Khatatbeh, Szimonetta Lohner, Imre Boncz, Dóra Endrei

**Affiliations:** 1 Doctoral School of Health Sciences, Faculty of Health Sciences, University of Pécs, Maria u. 5-7, Pécs, H-7621 Hungary; 2 Institute for Health Insurance, Faculty of Health Sciences, University of Pécs, Pécs, Hungary; 3 Cochrane Hungary, Clinical Center of the University of Pécs, Medical School, University of Pécs, Pécs, Hungary

**Keywords:** Performance, Evaluation, Assessment, Health, Hospital, Impact, Effect

## Abstract

**Background:**

Balanced Scorecard (BSC) has been implemented for three decades to evaluate and improve the performance of organizations. To the best of the researchers’ knowledge, no previous systematic review has performed a comprehensive and rigorous methodological approach to figure out the impact of BSC implementation in Health Care Organizations (HCO).

**Aims:**

The current work was intended to assess the impact of implementing the BSC on Health Care Workers’ (HCW) satisfaction, patient satisfaction, and financial performance.

**Methods:**

The authors prepared the present systematic review according to PRISMA guidelines. Further, the authors customized the search strategy for PubMed, Embase, Cochrane, Google Scholar databases, and Google’s search engine. The obtained studies were screened to isolate those measuring scores related to HCW satisfaction, patient satisfaction, and financial performance. The Risk of Bias (RoB) in the non-Randomized Intervention Studies (ROBINS-I) tool was used to assess the quality of observational and quasi-experimental studies. On the other hand, for the Randomized Controlled Trials (RCTs), the Cochrane (RoB 2) tool was used.

**Results:**

Out of 4031 studies, the researchers included 20 studies that measured the impact of BSC on one or more of the three entities (HCW satisfaction, patient satisfaction, and financial performance). Throughout these 20 studies, it was found that 17 studies measured the impact of the BSC on patient satisfaction, seven studies measured the impact on HCW satisfaction, and 12 studies measured the impact on financial performance.

**Conclusion:**

This systematic review provides managers and policymakers with evidence to support utilizing BSC in the health care sector. BSC implementation demonstrated positive outcomes for patient satisfaction and the financial performance of HCOs. However, only a mild impact was demonstrated for effects related to HCW satisfaction. However, it is worth noting that many of the studies reflected a high RoB, which may have affected the impacts on the three primary outcomes measured. As such, this systematic review reflects the necessity for further focus on this area in the future. Moreover, future research is encouraged to measure the real and current impact of implementing BSC in HCO during the pandemic since we did not find any.

**Supplementary Information:**

The online version contains supplementary material available at 10.1186/s12913-021-07452-7.

## Introduction

Since its development by Norton and Kaplan in 1992 [1], the Balanced Scorecard (BSC) has been utilized by many health care managers for the performance evaluation (P.E.) of Health Care Organizations (HCO) worldwide. Moreover, BSC can also be used as a strategic managerial tool by linking it to the organization’s strategy [2].

**Fig. 1:**
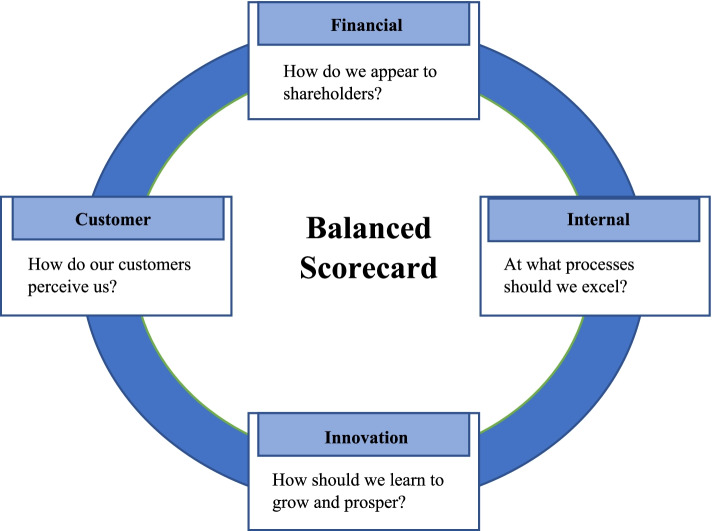
Balanced Scorecard Perspectives [1].

**Fig. 2:**
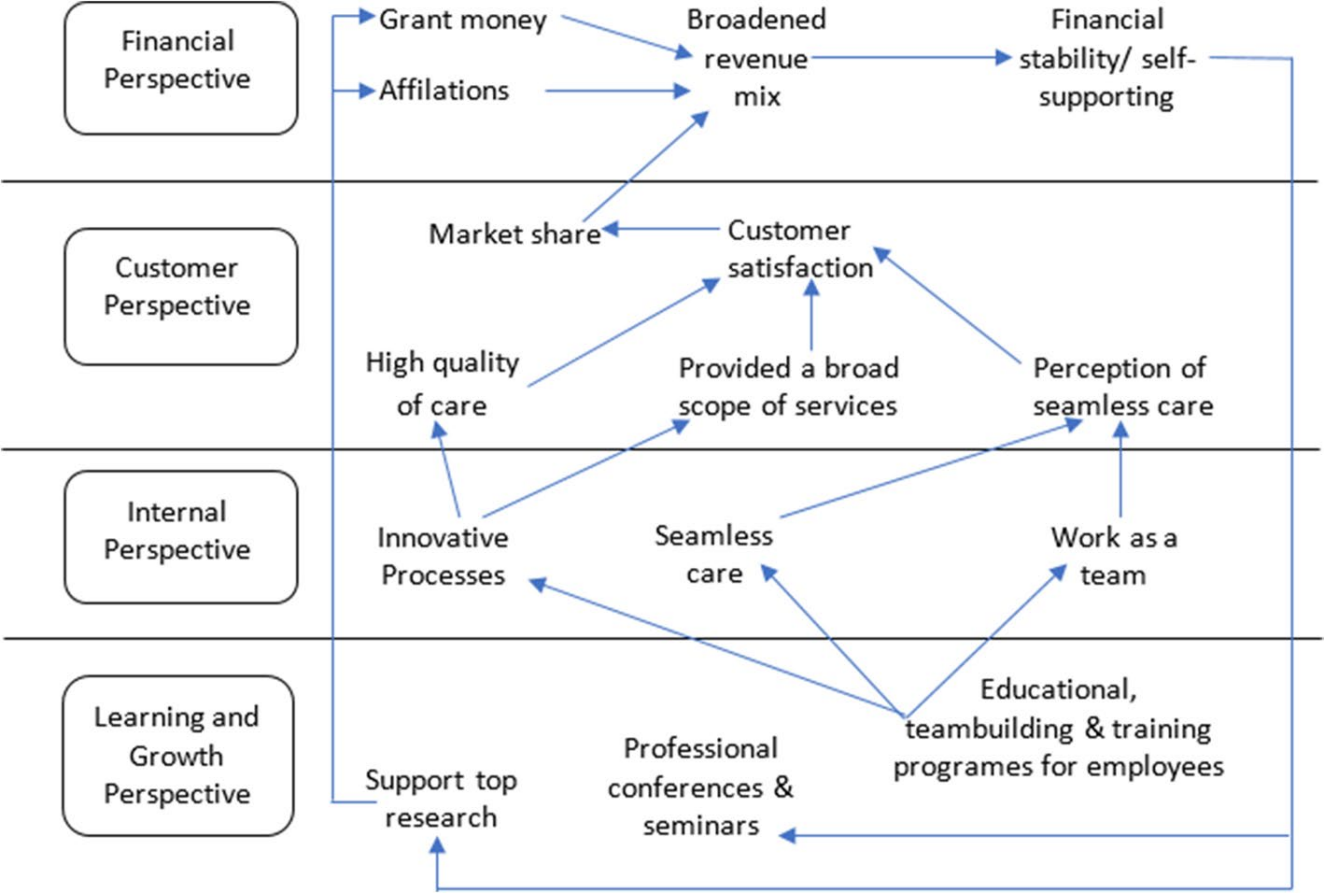
Duke University Health System Strategic Map [7]

The first generation of the BSC was used to evaluate four organizational perspectives: the financial perspective, the customers’ perspective, the internal processes’ perspective, and finally, the learning and growth perspective, all of which were steered by the organizational vision and strategy [1]. See Fig. 1. In this regard, it should be noted that a recent review re-categorized the BSC four perspectives for HCO into further 45 sub-dimensions or categories [3]. In the second generation of BSC, strategic maps were added to describe the cause-effect relationships between strategic objectives of each perspective [4]. In the third generation of BSC, destination statements, measures, and action plans were added to achieve the intended targets [5]. It is worth mentioning that Duke Children’s Hospital in the United States of America (USA) was the first HCO to implement BSC in 2000. As a result, the hospital was able to convert 11 million dollars of losses into four million dollars of profits [6]. See Fig. 2, which shows Duke University’s health system strategic map [7]. The BSC strategic maps show that the process flow of the cause-and-effect relationships ends with the customer and financial perspectives [4, 8].

More recently, the pandemic of Coronavirus Disease 2019 (COVID-19) imposed financial burdens on many countries and health care systems worldwide. In addition, the pandemic increased the psychological stress of patients and HCW [3, 9, 10]. Moreover, implementing tasks with particular standards and guidelines was vital in tackling the spread of COVID-19 [11]. However, conflicting managerial decisions and the lack of standardization capability were factors that brought dissatisfaction about HCO [12]. Consequently, it is deemed essential to evaluate previous BSC implementation’s effectiveness in HCO and determine if actual benefits remain to justify their continued use. It is worth noting that numerous systematic reviews regarding the impact of BSC implementation in non-health care-related fields, such as architecture [13], management, marketing, and accounting, already exist [14]. However, BSC reviews in health care only described the application of BSC or its perspectives [3, 15]. They lacked evaluation of the effectiveness of using BSC in HCO through systematically reviewed and evaluated literature. In this regard, only two reviews discussed the impact of BSC; one discussed the impact qualitatively [16], while the other mentioned a few examples of the positive impact [8]. To reiterate, this indicates that, until now, no comprehensive or rigorous methodological approach to assess the impact of BSC implementation in HCO has been recorded. Based on this gap in the literature, it was deemed essential to determine whether the previous BSC implementations at HCO were beneficial.

Thus, the present systematic review aims to gather all studies which have measured the impact of implementing BSC on HCW satisfaction, patient satisfaction, and financial performance at HCO; particularly, since these three attributes represent the latest affected perspectives in the strategic maps [4, 8]. Further, this review aims to assess and compare results among the included studies.

## Materials and methods

Our previous systematic review analyzed the dimensions and indicators of BSC utilized at the P.E. of HCO [3]. This systematic review was carried out by finding all studies that approached BSC implementation’s impact in HCO in adherence with the 27-point of the Preferred Reporting Items for Systematic Reviews and Meta-Analyses (PRISMA) checklist [17]. See (S1 Appendix).

### Eligibility criteria

The inclusion and exclusion criteria were set as shown in Table 1 below.

**Table 1:** Inclusion/Exclusion Criteria and Search Strategy for PubMed

PICO	Inclusion criteria	Exclusion criteria	Search Strategy (MeSH terms and keywords) for PubMed
**Population**	Any type of health care organization	Non- health organization	hospitals[MeSH Terms] hospital department[MeSH Terms] health[MeSH Terms]
**Intervention**	Performance assessment of health care organizations through implementing BSC	Studies that used other TQM tools such as MBNQA, ISO, SQA, six-sigma, etc.	“quality indicators, health care”[MeSH Terms] scorecard*[Text Word] “score card*”[Text Word]
**Comparator**	-Initiation of BSC implementation (at least one year of implementation) -Or: Comparing 2 measurements after BSC implementation for at least one year -Or: Gross change/ difference after at least one year of implementation	- Initiation of BSC implementation was in less than one year. -Gross change/ difference after less than one year -One-time measurement with no comparability.	No limitation was set in the search strategy, studies that measured BSC impact within less than one year of implementation were excluded after carefully examining the full texts.
**Outcome**	-Impact on financial indicators: profitability/loss, change in total revenues, change in total cost, ROI, ROA either in currency or in percentage. -Or: Impact on the patient satisfaction rate -Or: Impact on the HCWs’ satisfaction rate -The impact should be objective and measured/ quantitative.	-Impact on other indicators. -Number of patient complaints -HCWs’ burnout or turnover rate. -Cost/case or revenue/case change -Qualitative or subjective impact, for example: the managers’ opinions in impact	patient satisfaction[MeSH Terms] cost-benefit analysis[MeSH Terms] health care costs[MeSH Terms] Hospital personnel management[MeSH Terms] staff development[MeSH Terms] knowledge management[MeSH Terms] efficiency, organizational[MeSH Terms]
**Study design**	All study designs	_	No limitation regarding study design, type, or time was set in the search strategy

*Note*: *HCOs* Health Care Organizations, *BSC* Balanced Scorecard, *TQM* Total Quality Management, *MBNQA* Malcolm Baldrige National Quality Award, *ISO* International Organization for Standardization, *SQA* Singapore Quality Award, *ROI* Return On Investment, *ROA* Return On Assets

### Data sources, search strategy, and study selection

In the present systematic review, the search strategy was developed by the first, second, and fourth authors; because the first two authors are experts in health care management and BSC, and the fourth author is an expert in systematic reviews and meta-analysis. The search strategy was initially developed for the PubMed database based on the PICO (Population, Intervention, Comparison, and Outcome) tool [18], and depending on using both MeSH (Medical Subject Headings) terms and keywords. See (Table 1). Next, the strategy was adapted to Cochrane Central Register of Controlled Trials (CENTRAL), Embase, and Google Scholar databases, as per Cochrane’s recommendations [19]. The strategies developed for these databases can be found in (S2 Appendix).

The grey literature, pre-prints, and unpublished studies were searched on Google Scholar and Google’s search engine websites to reduce publication bias. Furthermore, the authors attempted to identify other potentially eligible studies or ancillary publications by searching the reference lists of any potentially eligible studies. The databases were searched until October 2020. Afterward, the first author conducted the search strategies on the electronic databases and removed the duplicates using the EndNote X9.2 program.

The first and second authors independently performed the selection of eligible studies. A discussion after each step was made or, if necessary, the third author was consulted for arbitration in case of disagreements. Initially, the titles and abstracts of the studies were examined to eliminate irrelevant studies. In the second step, the full texts of all potentially relevant studies were carefully reviewed to make a final decision based on the criteria mentioned above. Authors of studies with no available full texts or unclear impact duration were contacted to obtain further details and clarification.

### Data extraction and analysis

The following types of data were extracted from each of the final eligible studies: 1) author/s, year of publication, 2) country, 3) type of study, 4) duration of data collection 5) setting, 6) the number of health facilities, 7) number of participants, 8) data collection tool or data source, and 9) outcome (impact on patient satisfaction, HCW satisfaction, and financial performance). The data extraction was carried out independently between January and March 2021 by the first and second authors.

The research design of eligible studies was extracted directly from them. In case the research design was not explicitly mentioned, it was determined based on the role of the investigator of a given study. Specifically, if the BSC exposures were naturally determined and the investigator had no part, the study was considered observational. On the other hand, when the investigator assigned the BSC intervention, the study was deemed to be experimental.

The impact of BSC in eligible studies was explicitly mentioned or determined by calculating the difference between before and after implementation values. After that, the unification of the units was performed. Next, charts plotting for each outcome were performed by the first author and then reviewed by the first and second authors separately. If the impact measurement unit was not reported in the eligible studies, the authors of these studies were contacted. Lastly, all differences were compared, discussed, and judged by the two authors in the final step.

### Quality assessment

The Risk of Bias (RoB) assessment was performed by the first and second authors independently between March and June 2021 to assess the quality of the included studies. As per the Cochrane collaboration’s guidelines, the Cochrane (RoB 2) tool was used for the assessment of randomized controlled trials (RCTs) [20]. The Risk of Bias in non-Randomized Intervention Studies (ROBINS-I) tool was used to assess the observational and quasi-experimental studies [21]. As per the Cochrane Handbook, authors should avoid summarizing the overall RoB [22, 23]. Therefore, the RoB was analyzed at the study level and across studies.

In (RoB 2) tool, five types of bias were assessed: bias arising from the randomization processes, bias due to deviations from intended interventions, bias due to missing outcome data, bias in the measurement of outcomes, and bias in the selection of the reported results.

On the other hand, in the ROBINS-I tool, seven types of bias were assessed: bias due to confounding, bias in the selection of participants in a study, bias in measurement/classification of interventions/ exposures, bias due to deviations from intended interventions/ exposures, bias due to missing data, bias in the measurement of the outcomes, bias in the selection of the reported results.

While using the RoB 2 tool, each type of bias was assessed as low, high, or unclear. While using the ROBINS-I tool, each type of bias was evaluated into five categories: low, moderate, serious, critical, or no information. Afterward, the assessment results of the two reviewers were compared. Where there was disagreement, the fifth and sixth authors were consulted. Figures for RoB were prepared using the ROBVIS (Risk Of Bias VISualization) tool [24]. Lastly, it was recommended not to advocate quality appraisal as a criterion for inclusion in reviews [25]. Therefore, the authors decided to include all studies in this systematic review regardless of their quality assessment.

## Results

### Study selection

**Fig. 3:**
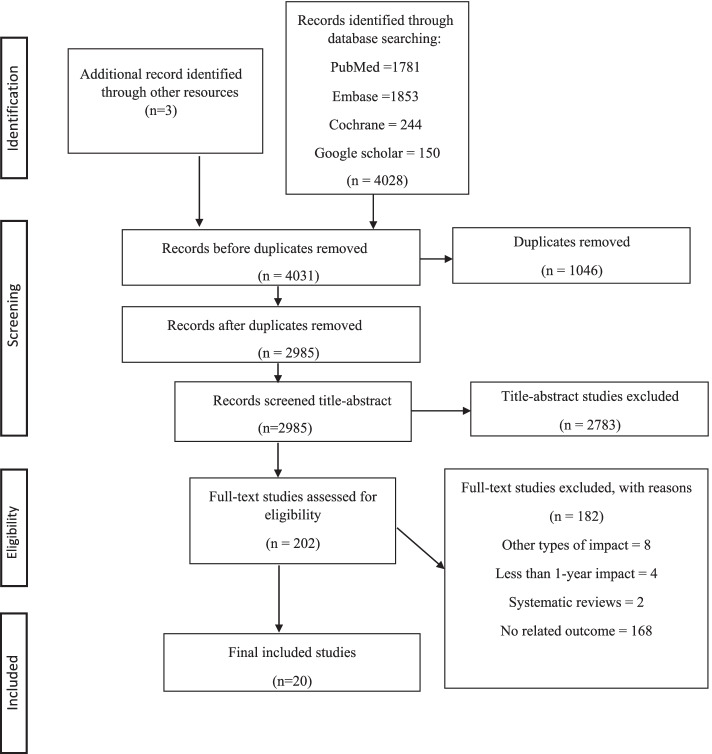
PRISMA Flow Diagram

Initially, the search strategy resulted in a total of 4031 studies. After removing the duplicates, a total of 2985 studies remained, which were screened based on their titles and abstracts. Then, irrelevant studies were excluded; thus, 202 studies remained. A careful examination of the included studies’ full texts was made; based on this, only 20 studies were finally included in the current systematic review. Details of the study selection process are shown in the PRISMA flow-chart (Fig. 3).

### Study characteristics

The main characteristics of the included studies are shown in Table 2.

**Table 2:** Summary of the Final Included Studies

Author/s, year of publication	Country	Design of the study	Duration of data collection	Setting	No. of health facilities	No. of participants	Data collection tool/ data sources
Harber, 1998 [26]	Canada	*Experimental* *uncontrolled pretest-posttest*	NR	Peel Memorial Hospital (Hospital in general + Laboratory)	1	NR	NR
Meliones, 2000 [5]	The USA- North Carolina	*Experimental* *uncontrolled Interrupted time series*	1996–2000	Duke Children Hospital	1	NR	NR
Pink et al., 2001 [27]	Canada	*Observational prospective longitudinal*	1997–1998	Markham Stouffville hospital	1	NR	Surveys + hospitals’ reports
Gumbus et al., 2003 [28]	USA	Case Study/ *observational retrospective longitudinal*	1999–2001	Bridge port hospital	1	NR	The patient satisfaction measurement system
Smith & Kim, 2005 [29]	USA	*Observational prospective and retrospective longitudinal*	2001–2004	Summa’s Food & Nutrition Service Department at Summa Health System (STH & ACH hospitals)	2	NR	Press Ganey’s standard inpatient survey + audit checklists
Devitt et al., 2005 [30]	Canada	*Observational prospective and retrospective longitudinal*	2004–2005	Toronto East General Hospital	1	NR	Data extraction from hospital records
Yang & Tung, 2006 [31]	Taiwan	Retrospective longitudinal/ o*bservational*	2000–2002	General hospitals & their supervisor agency	21	NR	Secondary data from the department of health + primary data structured questionnaire measuring hospitals’ organizational learning and growth perspective
Lorden et al., 2008 [32]	NR	Multimethod quantitative and qualitative case study/ *experimental* *uncontrolled Interrupted time series*	Jan, 1998- June, 2004	Community hospital	1	300 Inpatient/quarter, 700 outpatient/quarter, 227 employees (1st survey), 191 employees (2nd survey)	Employee satisfaction survey + patient satisfaction survey (via email)
Josey & Kim, 2008 [33]	USA-Ohio	*Observational retrospective longitudinal*	2006	Barberton Citizens hospital (BCH)	1	NR	Patient satisfaction survey
Chang et al., 2008 [34]	Taiwan	*Observational retrospective longitudinal*	2001–2005	Mackay Memorial Hospital	1	NR	NR
Hansen et al., 2008 [24]	Afghanistan	*Observational retrospective longitudinal*	July to October of (2004/2005/2006)	Health facilities	> 600	1700 HCWs, 5800 patients- provider interaction	NR
Chu et al., 2009 [35]	Tawian	Case study/ *experimental* *uncontrolled Interrupted time series*	2004–2006	The nursing department at a public teaching hospital in Taiwan	1	13 reference nurses’ group	Financial data from hospital + questionnaires to executives (the weights of indicators)
Edward et al., 2011 [36]	Afghanistan	*Observational retrospective longitudinal*	2004–2008	Health facilities in Afghanistan	700	1500 HCWs, 5000 patients	National health services performance Assessment + interviews with patients and HCWs
Fields & Cohen, 2011 [37]	USA	*Experimental uncontrolled Interrupted time series*	2009–2010	Oregon Health and science university family medicine (Clinics)	1	NR	Press Ganey survey for patient satisfaction + medical records.
Koumpouros, 2013 [38]	Greece	Case study/ *experimental uncontrolled Interrupted time series*	18 months but not specified when exactly	General Panarcadian Hospital of Tripolis	1	NR	Questionnaires and interviews
Smith et al., 2014 [39]	Canada/ Alberta	*Experimental* *uncontrolled pretest-posttest*	2010–2011 (12-month trial), March 31, 2013 (results)	Hospitals in Alberta including hip and knee surgeries	12	NR	NR
Abdullah et al., 2014 [40]	Indonesia	Cross-sectional/*observational prospective and retrospective longitudinal*	April–December, 2013	Cibto Mangunkusumo Hospital- Digestive endoscopy center	1	76 patients	Endoscopy reports + interviews based on structured questionnaires
Mutale et al., 2014 [25, 41]	Zambia	Cluster randomized intervention/ *experimental RCT*	2011–2013	Health facilities in Zambia	12	96 HCWs, 429 patient interviews, 410 patient observations	A survey in facilities+ interviews with HCWs and patients + patient observation + survey with households
Catuogno et al., 2017 [42]	Italy	Case study/ *Experimental uncontrolled pretest-posttest*	2007–2008, & 2014–2015	Hematology department at a Research hospital in Italy	1	14	Stakeholder satisfaction; questionnaires + care processes + hospital discharge report + charity report + research process + departmental report + economic and financial; hospital discharge database + departmental report + charity report
Widyasari & Adi, 2019 [43]	Indonesia	Descriptive Quantitative longitudinal/*observational prospective and retrospective*	During the year 2018	Bali Mandara Hospital of Bali (Governmental hospital)	1	30	Participant observation + structured interviews + semi-structured interviews + documentation.

*Note*: *NR* Not Reported, *HCWs* Health Care Workers. *Italic* are designs based on our classification but not reported.

#### Location/ country

Regarding the implementation location, nine studies were implemented in North America, two in Europe, one in Africa, seven in Asia, and one did not specify the location. It should be noted that 14 studies were performed in high-income countries, two in upper-middle-income countries, one in a lower-middle country, and only two in low-income countries.

#### Setting

Out of the 20 selected studies, 16 were performed in hospitals or hospital departments, and four in health care facilities or clinics. See Table 2.

#### Language

Even though no limitation was imposed on language, all of the selected 20 studies measuring the impact of BSC implementation were written in English.

#### Study designs

Out of the 20 selected studies, only three studies reported their study designs explicitly. However, our classification showed that 11 studies were observational since the investigators were not involved in implementing BSC; instead, these investigators only observed the results of already implemented BSCs at HCO. On the other hand, the remaining nine studies were experimental. One out of the nine was RCT, while the other eight were quasi-experimental studies, which included three pretest-posttest components and five Interrupted Time Series. See Table 2. Notably, only three studies [32, 36, 37] randomly selected HCO, participants, or both.

#### Variations regarding the data collection instruments

Variances among the data collection instruments used in the 20 studies are shown in Table 2. Notably, the employed instruments were validated only in six studies [27, 28, 32, 35, 39, 42]. Additionally, only five studies [27, 28, 39, 42, 43] assessed the instruments’ feasibility. The pre-testing of the instruments was carried out only in three studies [27, 32, 35]. In addition, only five studies [27, 31, 38, 42, 43] assigned weights for the indicators or assessed their importance before implementation. Further, only one study [29] evaluated the indicators depending on more than one source for the same variable.

#### BSC generations

The 20 studies chosen for this systematic review utilized different BSC generations. The first generation of BSC was employed in seven studies [28–31, 33, 39, 42] which discussed explanations, the definition of perspectives and indicators, and how to measure each indicator. Besides these seven studies, one other study [34] used the first generation BSC; however, only customer and patient satisfaction were explained in the way they were measured. Further, only five of the 20 studies [27–30, 33] specified the source for each perspective/indicator, while one study [42] mentioned them partially.

The aspects of BSC’s second-generation were found in five of the 20 studies [26, 28, 29, 31, 34], where users modified the objectives of each indicator during implementation to suit strategy, vision, mission, and goals. Additionally, two other studies [42, 43] modified these objectives partially but failed to explain them sufficiently. Further, strategic maps were only illustrated in six studies [26, 28, 31, 33, 38, 40]. Finally, it is worth noting that only three studies [28, 29, 31] displayed the cause-effect cascade between indicators and targets.

Regarding the third generation’s aspects, seven of the 20 studies [26, 28–31, 33, 34] approached destination statements or targets within a time horizon. Besides, one study [6] approached the length of stay indicator only. Additionally, only one study [26] approached strategic initiatives or action plans to achieve the targeted performance.

#### BSC’s impact type

**Fig. 4:**
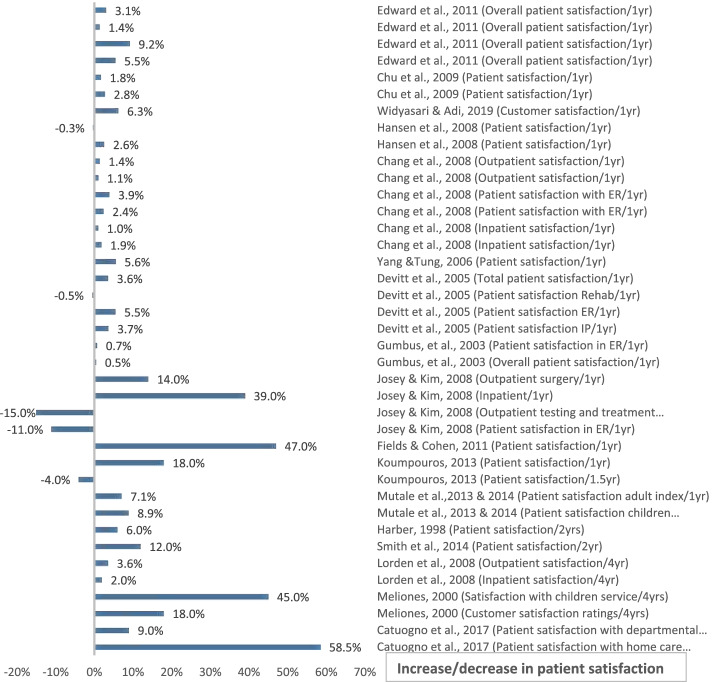
Patient Satisfaction Impact. Increase or decrease in patient satisfaction rate after BSC implementation (%)

**Fig. 5:**
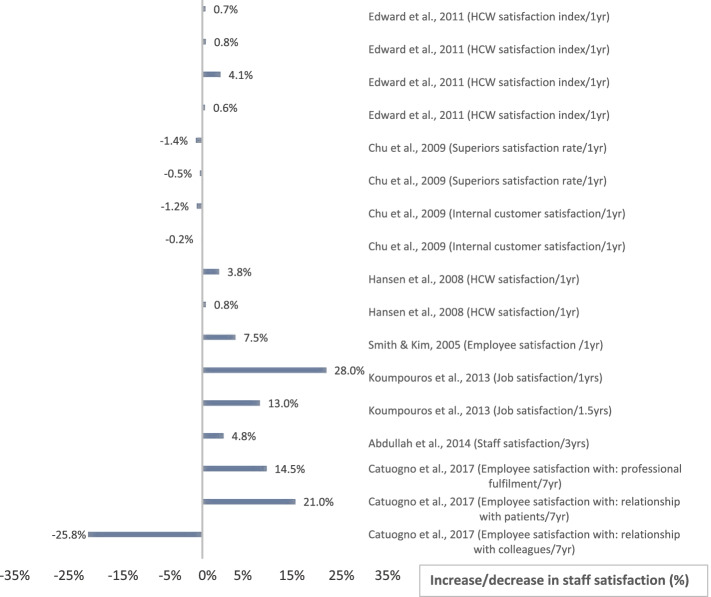
HCW Satisfaction Impact. Increase or decrease in HCW satisfaction rate after BSC implementation (%)

**Fig. 6:**
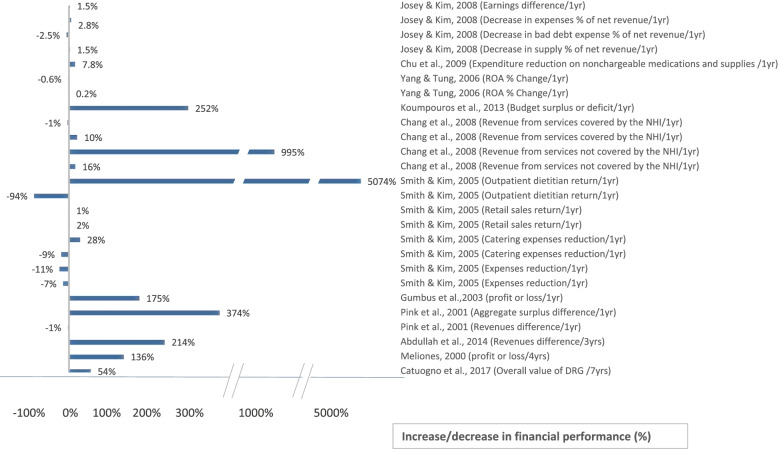
Financial Impact (%). Increase or decrease in financial performance after BSC implementation (%)

**Fig. 7:**
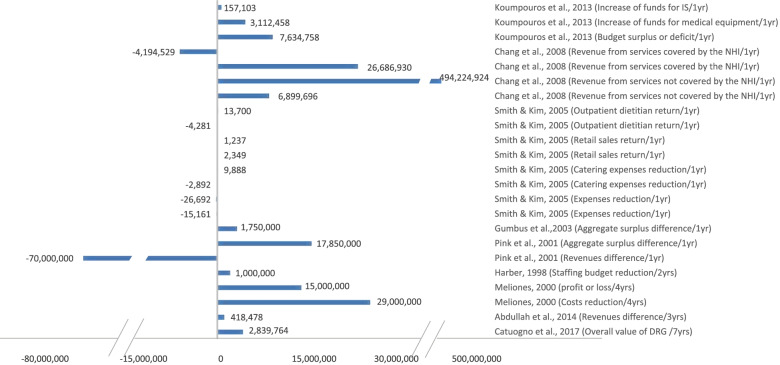
Financial Impact (USD). Increase or decrease in financial performance after BSC implementation (USD)

The included studies assessed different outcomes for implementing BSC. Out of the final 20 eligible studies, 17 studies [6, 26–29, 31–38, 42–44] measured the impact of BSC on patient satisfaction, 7 measured HCW satisfaction [27–30, 36, 37, 45], and 12 studies measured financial performance [6, 27–30, 33, 34, 40, 41, 43–45]. However, the measured variables varied among studies, even in terms of the same dependent variable (Figs. 4, 5, 6, 7). For example, BSC’s impact on patient satisfaction varied from overall satisfaction to the satisfaction of specific categories, such as adults, children, inpatients, outpatients, patients in the emergency room, patients in rehabilitation. In addition, the measured variables varied based on the service type, such as satisfaction of home care services and departmental services.

Regarding HCW satisfaction, the name assigned to the targeted population varied from staff, employees to HCW. Further, the HCW satisfaction type varied, for instance, from HCW satisfaction towards their job to HCW satisfaction towards their superiors. However, the financial variable had the greatest variation among all three primary outcomes measured. Specifically, it was found that there exists a reduction in costs, expenditures, HCW budget, expenses, catering expenses, expenses/net revenues, bad debt expenses per net revenue, and supply per net revenue. On the other hand, an increase in revenues types included; returns, profits, aggregate surplus, funds, the value of drug-related groups, and return on assets.

Moreover, the unit used for financial impact assessment differed among studies. For example, all studies used currencies for assessment, where these currencies also varied between studies, except for a few studies [27, 33, 34] which used a percentage method. As an attempt to reduce bias, all currencies were converted to United States Dollar to standardize and make the comparison across studies more consistent regarding the financial outcomes in the systematic review. Further, the authors of one study [28] were contacted for clarification since they did not report the currency. As a result, Figs. 6 and 7 were designed as seen below; one for the impact in currencies and the other for the impact in percentages.

Most studies used a percentage score to measure the impact on patient and HCW satisfaction, except for three studies [28, 30, 45], which performed the measurement based on a four or five point-Likert scale. However, to make the comparison consistent, all Likert scales were converted to percentages (scores out of 100%). It should be noted that only two of the 20 studies [35, 45] discussed the statistical significance of the results. Hence, the magnitude of change (in percentage) was taken into consideration in our analysis.

#### Time of measuring the outcomes

To make an objective comparison and avoid falling into bias, we reported the time between implementing BSC and assessing its impact in the included studies. Further analysis demonstrated that the intervals between the initiation of BSC implementations and the measurements of impacts varied among studies. In particular, one study [29] reported the results based on 18 months of implementation, two studies [38, 44] took 2 years, one study [45] took 3 years, two studies [6, 35] took 4 years, and one study [28] took 7 years of implementation. The remaining 13 studies reported results based on 1 year of implementation. Due to the previously mentioned variations in measured variables, duration of implementing BSC, and differences in data collection instruments or data sources, refer to (Table 2), the authors decided that conducting a meta-analysis would not lead to meaningful results. Instead, a comparison of the impact was performed using the bar charts. See (Figs. 4, 5, 6, 7).

### The impact of BSC implementation

The outcomes of BSC implementation in each of the 20 included studies are shown above in (Figs. 4, 5, 6, 7), which illustrate the impact on patient satisfaction, HCW satisfaction, and financial performance (in percentage and currency). The exact impact type and duration were specified for each measurement, and the results are discussed further in the discussion section below.

Since we did not limit our search strategy to the three BSC impacts defined in our aim, eight studies of different impact types resulted in the abstract screening process. Examples of the other BSC impact types include; influencing the market share, the number of new patients, the number of consultations and visits, community satisfaction, percentage of residents receiving outreach activities, number of sampled children, availability of essential drugs, and decreasing the average length of stay [46, 47]. However, after reading the full texts, the authors decided to exclude these studies as they lacked sufficient relevance to our research aim. See (S 3 Appendix).

### Quality assessment

As illustrated in S4 Appendix, each study was evaluated in terms of RoB. For that purpose, the RoB 2 tool was employed to assess the sole RCT study [32], in which the assessment was deemed fair, except for the performance bias. On the other hand, the RoB in the quasi-experimental and observational studies was measured using the ROBINS-I tool; and it was found that there was no information about analysis methods of confounders’ adjustments except in four studies [33, 36, 39, 42]. The confounding agents were apparent in three studies [27, 35, 37]. However, the three studies failed to adjust for the confounders, which may have affected the precision of the measurement.

Furthermore, the selection bias across studies reflected serious RoB in five studies [27, 30, 31, 33, 40]. A possible reason the intervention and the follow-up did not coincide together and a potentially substantial amount of follow-up time was missing in the analyses. The moderate risk of bias showed that the intervention status was well defined, but some aspects of the assignments of intervention status were determined retrospectively. Further, outcome measurements bias was raised either due to the non-blinding of intervention among assessors [37] or because the outcome measure was subjective and likely to be influenced by other factors [29, 38]. See (S4 Appendix).

## Discussion

### Discussion of the main results

This systematic review aimed to identify all the studies which measured the impact of BSC implementation on three variables: HCW satisfaction, patient satisfaction, and financial performance at HCO, and then proceeded to analyze the effect of these BSC implementations.

The analysis of the results reflected a remarkably positive impact of BSC on patient satisfaction in most studies. The same positive impact of BSC implementation holds for financial performance in both currency and percentage indicators. Notably, the authors found that almost all studies showed a positive impact, amounting to several million dollars. However, a few studies reflected a moderately negative impact on financial performance, which form three distinct categories. The first category includes study [29], which explained the occurrence of unintended events that may have negatively affected financial performance. The second category comprises studies [30, 40] that revealed a highly positive impact on financial performance in previous or subsequent years, which may reflect a sloth in following up. The third category includes studies [28, 39] that showed a positive impact on financial performance on one or more of the other impact types. On the other hand, the analysis of BSC impact for HCW satisfaction revealed a less remarkably positive impact. See (Figs. 4, 5, 6, 7).

### Agreements and disagreements with other studies or reviews

The findings obtained from the present systematic review are in line with a systematic review [14] that reviewed BSC’s benefits in business, management, and accounting fields. Furthermore, the present study is the first to summarize all BSC implementations and their impacts on the health care sector based on quantitative comparisons. Moreover, the current study was compared with other reviews in the health care sector. For instance, a review [15] carried out a mere description regarding the application of BSC. In contrast, a review [3] only summarized the perspectives and dimensions utilized. Lastly, a review [16] only mentioned examples about BSC impact.

One probable explanation for the mild impact on HCW satisfaction can be referred to the lack of managerial engagement with the non-managerial HCW upon BSC implementation, the lack of understanding by HCW about the advantages of BSC implementation s, or the fear of potential responsibility and accountability placed upon HCW due to BSC implementation. As a result, HCW may have declined to implement BSC, contributing to a lower satisfaction score. In conclusion, future researchers should consider increasing employee participation in BSC implementations.

For instance, in a study [29], the employees did not have incentives or motives to participate in BSC since they were permanent employees. Further, the study showed that HCW above 40 years old negatively influenced creativity and productivity upon BSC implementation. Other researchers in [48] also referred to this challenge, who noted that major deficiencies arose from qualified personnel and HCW aging. However, those researchers have also suggested that the high-ranking qualifications of HCW, driving learning and a growth perspective, will eventually generate motivation for new HCW to resolve this issue. Other proposed ideas to solve this problem were creating an open environment for learning and growth and encouraging active communication with HCW to ensure the successful implementation of BSC. Other researchers [49] encouraged senior management commitments to involve non-managerial HCW, promoting clear articulation of benefits and relevancy of BSC to clinicians. This challenge mirrors the findings of another review [50], which realized that the attitude perceived by health care professionals of accreditation was negative and skeptical because of quality concerns regarding services and their cost. Therefore, the authors in the latter study suggested that health care professionals, especially physicians, require more intensive education about the potential benefits of accreditation.

Finally, the quality assessment revealed that many studies had high RoB, which may have affected the impact results. A recommendation for the researchers and managers implementing BSC in the future is to dedicate more focus to raising the quality of implementation and lowering the RoB. Moreover, a better focus on the second and third generations of BSC aspects is essential.

### Strengths and weaknesses

The current systematic review contains several strengths. To our knowledge, this is the first paper that has analyzed all the studies which measured the impact of BSC on patient satisfaction, HCW satisfaction, and financial performance in HCO. The results and analysis of this systemic review support a positive impact for applying BSC in HCO, especially on patient satisfaction and financial performance. Further, a greater emphasis on the role of HCW is required when implementing BSC since HCW satisfaction showed slightly positive, almost zero, or somewhat negative scores in most studies included.

Additionally, the three primary outcome measures concentrated upon in this systematic review are considered the last destination for impact in the strategic maps and the causal effects at most BSC studies. Finally, unlike other BSC reviews [8, 16], which included definitions of biobanks, pharmacies, laboratories, radiology, and medical colleges in HCO, this review limited the definition to the primary, secondary, or tertiary health care organizations. This strategy leads to the homogeneity of the resulting studies and leads to more valid comparisons among the results.

Nevertheless, this paper has some limitations. First, it focused on the impact of BSC on the three chosen indicators only, whereas impacts on other types of indicators were not considered for analysis. Due to the vast variations of indicator types, analysis of these indicators presents a challenge, requiring narrowly specified modes of analysis. Secondly, no meta-analysis could be applied to this systematic review resulting from the heterogeneity of studies regarding their data collection tools and the enormous variation in the types of indicators. However, the later variation was clarified in the charts, and the data collection tool was specified for each study. Thirdly, the current review included studies that measured the impact after at least 1 year of implementation. Fourthly, it is essential to mention that the impact comparability is roughly more rational for patient satisfaction and HCW satisfaction than financial performance. This could be referred to as the comparison ability based on a percentage score of 100 for the satisfaction variables. Additionally, the change in financial performance based on currency could be influenced by other confounding factors such as the HCO size or the number of health facilities included in the study. Therefore, future studies should consider these confounding factors. Moreover, future studies should reduce the RoB due to the lack of high-quality BSC implementations in the literature. Finally, this review searched for the BSC implementation in health care databases; consequently, future systematic reviews are recommended to include studies in management and health policy databases.

## Conclusions

In conclusion, this systemic review offers evidence to HCO and policymakers on the benefits of implementing BSC in HCO. Although the quality assessment revealed that many studies had a high RoB, BSC implementation positively influenced HCO patient satisfaction and financial performance. Based on the findings in the present review, researchers are encouraged to focus on lowering the risk of bias in BSC implementation in the future. HCO managers are also advised to consider HCW satisfaction and engagement with BSC implementations. Finally, an additional assessment of the BSC impact in HCO during the COVID-19 pandemic is required, as we could not find any.

## Supplementary Information

**Additional file 1: S1 Appendix. PRISMA checklist.** 27-point checklist of the Preferred Reporting Items for Systematic Reviews and Meta-Analyses (PRISMA) checklist.

**Additional file 2: S2 Appendix.** Search strategies in PubMed, Embase, Cochrane, and Google scholar.

**Additional file 3: S3 Appendix.** Data set of title/abstract and full-text screenings.

**Additional file 4: S4 Appendix.** Risk of Bias.

## Data Availability

All data generated or analyzed during this study are included in this published article [and its supplementary information files].
