# Correction to: The deployment of balanced scorecard in health care organizations: is it beneficial? A systematic review

**DOI:** 10.1186/s12913-022-07555-9

**Published:** 2022-02-10

**Authors:** Faten Amer, Sahar Hammoud, Haitham Khatatbeh, Szimonetta Lohner, Imre Boncz, Dóra Endrei

**Affiliations:** 1 Doctoral School of Health Sciences, Faculty of Health Sciences, University of Pécs, Maria u. 5-7, Pécs, H-7621 Hungary; 2 Institute for Health Insurance, Faculty of Health Sciences, University of Pécs, Pécs, Hungary; 3 Cochrane Hungary, Clinical Center of the University of Pécs, Medical School, University of Pécs, Pécs, Hungary

**Correction to: BMC Health Serv Res 22, 65 (2022)**

**https://doi.org/10.1186/s12913-021-07452-7**

Following publication of the original article [1], the authors identified that the arrows were misplaced in Fig. 2 due to a format conversion issue. The correct figure is given below.

**Fig. 2:**
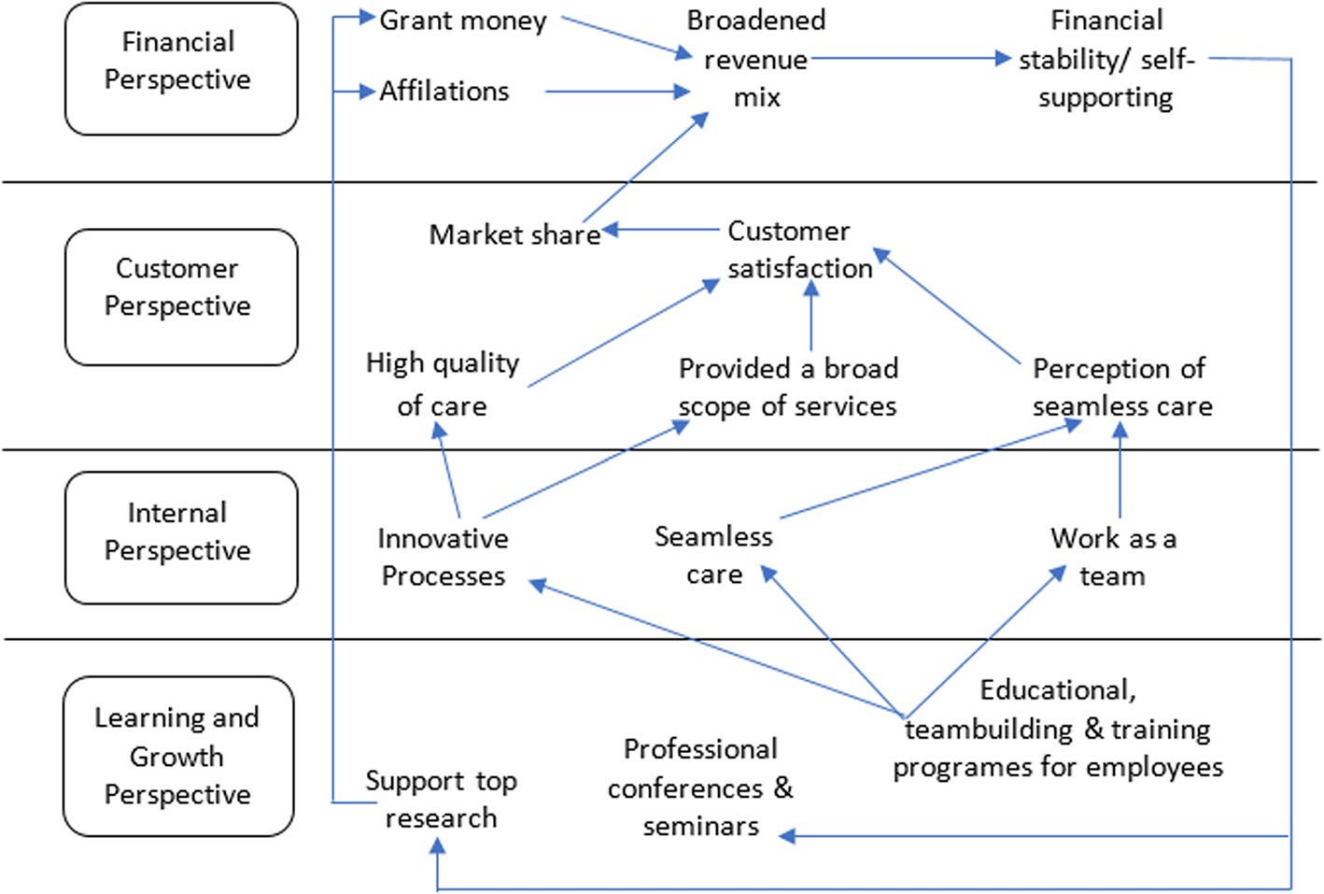
Duke University Health System Strategic Map [7]

The original article has been corrected.
