# Supplementary material for: The deployment of balanced scorecard in health care organizations: is it beneficial? A systematic review
Source: BMC Health Serv Res. 2022 Jan 13;22:65. doi: 10.1186/s12913-021-07452-7 (PMC8758212; doi:10.1186/s12913-021-07452-7)

Risk of Bias using ROBINS-I for Non-Randomized Studies of Interventions (NRSI) studies (Quasi- experimental and obdervational):


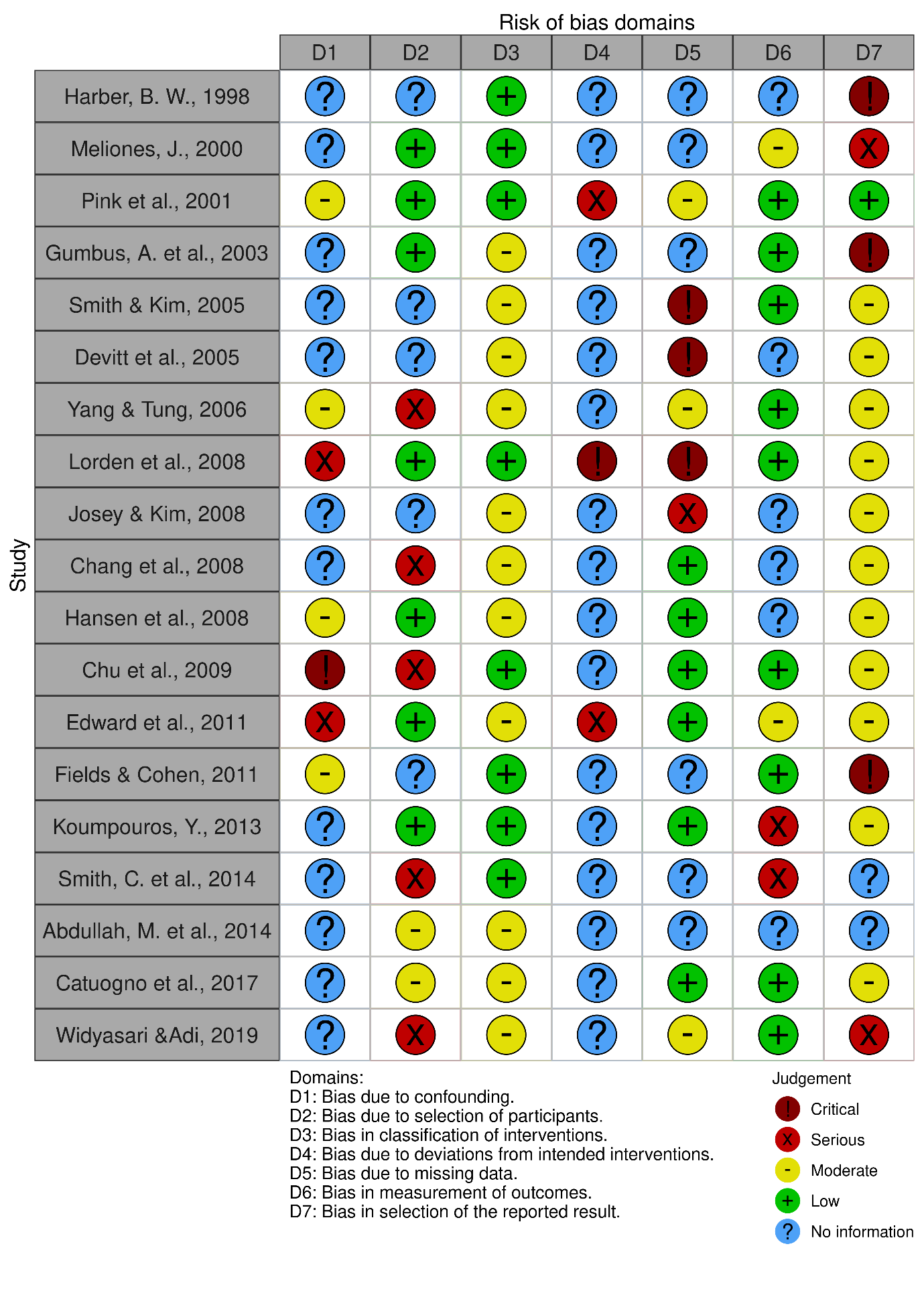


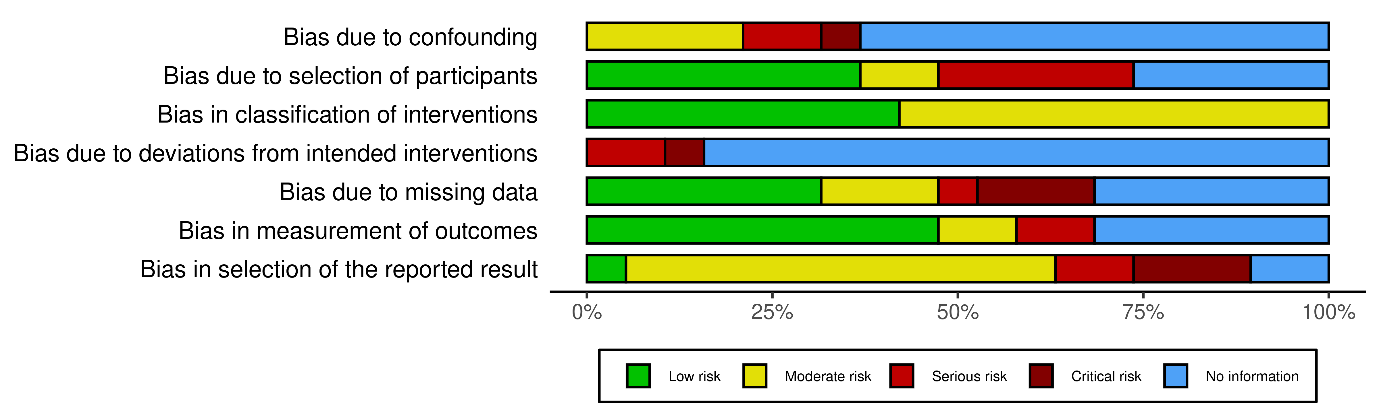


Risk of bias using ROB 2 for Randomized Controlled Trials (RCT) studies:


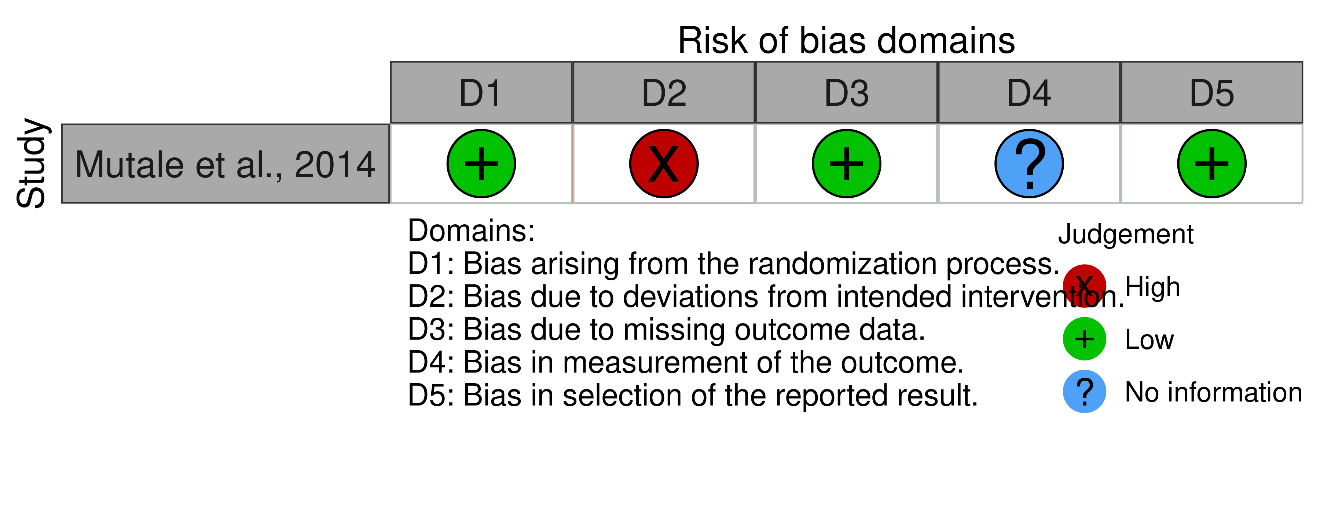

Supplement: Supplementary file 3 — Additional file 3: S3 Appendix. Data set of title/abstract and full-text screenings. [file 12913_2021_7452_MOESM3_ESM.docx]
